# Irradiation of *Nf1* mutant mouse models of spinal plexiform neurofibromas drives pathologic progression and decreases survival

**DOI:** 10.1093/noajnl/vdab063

**Published:** 2021-04-23

**Authors:** Danny Laurent, Abbi E Smith, Waylan K Bessler, Marc Mendonca, Helen Chin-Sinex, Martina Descovich, Andrew E Horvai, D Wade Clapp, Jean L Nakamura

**Affiliations:** 1 Department of Radiation Oncology, School of Medicine, University of California, San Francisco, San Francisco, California, USA; 2 Department of Pediatrics, Indiana University School of Medicine, Indianapolis, Indiana, USA; 3 Department of Radiation Oncology, Indiana University School of Medicine, Indianapolis, Indiana, USA; 4 Department of Pathology, School of Medicine, University of California, San Francisco, San Francisco, California, USA

**Keywords:** heterozygosity, malignant transformation, Neurofibromatosis I, plexiform neurofibromas, radiation

## Abstract

**Background:**

Genetically susceptible individuals can develop malignancies after irradiation of normal tissues. In the context of therapeutic irradiation, it is not known whether irradiating benign neoplasms in susceptible individuals promotes neoplastic transformation and worse clinical outcomes. Individuals with Neurofibromatosis 1 (NF1) are susceptible to both radiation-induced second malignancies and spontaneous progression of plexiform neurofibromas (PNs) to malignant peripheral nerve sheath tumors (MPNSTs). The role of radiotherapy in the treatment of benign neoplasms such as PNs is unclear.

**Methods:**

To test whether radiotherapy promotes neoplastic progression of PNs and reduces overall survival, we administered spinal irradiation (SI) to conditional knockout mouse models of NF1-associated PNs in 2 germline contexts: *Nf1*^*fllfl*^*; PostnCre*^*+*^ and *Nf1*^*fl/-*^*; PostnCre*^*+*^. Both genotypes develop extensive *Nf1* null spinal PNs, modeling PNs in NF1 patients. A total of 101 mice were randomized to 0 Gy, 15 Gy (3 Gy × 5), or 30 Gy (3 Gy × 10) of spine-focused, fractionated SI and aged until signs of illness.

**Results:**

SI decreased survival in both *Nf1*^*fllfl*^ mice and *Nf1*^*fl/-*^ mice, with the worst overall survival occurring in *Nf1*^*fl/-*^ mice receiving 30 Gy. SI was also associated with increasing worrisome histologic features along the PN-MPNST continuum in PNs irradiated to higher radiation doses.

**Conclusions:**

This preclinical study provides experimental evidence that irradiation of pre-existing PNs reduces survival and may shift PNs to higher grade neoplasms.

**Key Points**

- *Nf1* ^*fllfl*^ *; PostnCre* ^*+*^ and *Nf1*^*fl/-*^*; PostnCre*^*+*^ mice are robust models of plexiform neurofibromas.
- Focal, fractionated irradiation of pre-existing spinal plexiform neurofibromas in *Nf1* mutant mouse models reduces survival.
- Irradiation of plexiform neurofibromas is associated with higher grade histopathologic features on the continuum between plexiform neurofibromas to MPNSTs.

**Importance of the Study**

The role of radiation therapy in the management of plexiform neurofibromas arising in individuals with NF1 is unclear with concerns for malignant transformation. Contributing to the controversy is the question as to whether radiotherapy to plexiform neurofibromas can transform these benign neoplasms into higher grade tumors. This is the first study that tests whether therapeutic spinal irradiation in preclinical mouse models of spinal plexiform neurofibromas impacts survival and malignant transformation. Our experimental insights inform a controversial issue with clinically relevant and translatable correlates.

Radiation therapy is an integral part of cancer treatment; however, ionizing radiation (IR) is also a mutagen, capable of inducing cancer in most tissues at all ages.^1^ Exposure to IR causes single- and double-strand DNA breaks, which can lead to genomic instability, mutations, and subsequent malignant transformation of cells. Understanding the mechanisms and relationships between IR and carcinogenesis from healthy cells, as well as benign tumor cells with oncogenic characteristics, into aggressive cancers is needed to minimize risks while maintaining treatment efficacy.

Neurofibromatosis type 1 (NF1) is a complex autosomal disorder caused by germline mutations in the *NF1* gene, affecting approximately 1 in every 3000 individuals.^2^ Individuals with NF1 are predisposed to developing tumors originating from the embryonic neural crest.^3^ Among these typically benign tumors are plexiform neurofibromas (PNs), which are characterized by differentiated Schwann cells in a varied microenvironment composed of perineural-like cells, fibroblasts, vascular, and mast cells. PNs develop in approximately 30%–50% of NF1 patients.^4^ In 10% of NF1 patients, PNs can transform further into malignant peripheral nerve sheath tumors (MPNSTs), representing a severe complication of the NF1 syndrome.^4^ The majority of MPNST cases (40%–50%) occur in individuals with NF1 syndrome and the remainder arise sporadically (40%–47%) or following radiotherapy (10%–13%) as therapy-induced malignancies.^5,6^

Radiotherapy is a standard treatment for MPNST, and known to provide superior nerve-sparing and improve functional outcomes of patients. Radiotherapy is used with reservation to treat PN in NF1 patients due to concerns about radiation-induced malignancies. The concern for radiotherapy uses in NF1 patients is justified, given recent epidemiologic evidence indicating that radiotherapy is associated with development of subsequent neoplasms^7^ resulting from irradiation of normal tissues. Individuals with NF1 are at risk for developing second malignancies after radiotherapy.^7^ Epidemiological investigation of NF1 childhood cancer survivors showed that the cumulative incidence of subsequent neoplasms was 7.3%, with this risk being 2.4-fold higher than in non-NF1 cancer survivors.^7^ Further, the risk of subsequent neoplasm increases as time since primary diagnosis increases.^1^ A related issue is whether radiotherapy to benign tumors in NF1 patients is similarly associated with transformation risk and therefore significantly worse clinical outcomes. Because this question cannot realistically be answered in clinical practice, we developed mouse models to test radiation effects and clinical outcomes.

We previously developed mouse models of radiation-induced malignancies that replicate clinical radiotherapy parameters, by delivering total body irradiation,^3^ and focal fractionated irradiation to the cranium^8^ and abdomen,^9^ similar to clinical radiotherapy. In this work, we tested whether increasing doses of radiotherapy promote malignant transformation of PN to higher grade tumors, hypothesizing that increasing doses of radiotherapy are associated with a decrease in survival and an increase in the aggressiveness of tumors. We developed a mouse model of focal spinal irradiation (SI) to PN, which recapitulates clinical radiotherapy with regard to anatomic localization and dosing scheme. We tested *Nf1*-driven PN development in 2 different genetic backgrounds, *Nf1* heterozygous and wildtype, to test whether radiation effects on PNs is dependent on the background’s *Nf1* status. Our findings indicate that SI is associated with decreased survival in a dose-dependent manner. In addition, background heterozygosity for *Nf1* was associated with a significantly worse survival after SI compared to *Nf1* wildtype background, suggesting that the *Nf1* mutant background negatively influences survival after SI. Together, these findings point to genetic background effects on survival and tumor progression after spinal irradiation and directly inform clinical considerations in the treatment of patients with NF1.

## Materials and Methods

### Ethical Guidelines Regulations

All work was performed under a research protocol approved by the University of California, San Francisco (UCSF) Committee on Human Research (IRB protocol 11-07304).

### Mouse Cohort and Treatment

Animal experimentation was performed under an approved Indiana University School of Medicine IACUC protocol (#11405). The experimental cohort was composed of 101 male mice with 2 distinct genotypes, *PeriCre*^*+*^; *Nf1*^*fl/fl*^ and *PeriCre*^*+*^; *Nf1*^*fl/-*^. Mice were maintained in a mixed background (C57BL/6 and 129/S). Mice were assigned to one of the following 3 SI treatments: 0 Gy, 15 Gy (5 daily doses of 3 Gy), and 30 Gy (10 doses of 3 Gy). SI was administered one fraction per day, 5 times per week (schematic shown in Supplementary Figure S1). These radiation doses and frequency were chosen based on prior work indicating the general safety of these regimens, which enables long-term follow-up of irradiated animals,^8,9^ and to approximate clinically used radiation fractionation schemes. Mice were monitored for 12 months following SI or until they develop signs of systemic illness that necessitated euthanasia. The 12-month monitoring period was chosen based on the average latency period for the development of soft-tissue sarcoma following total body irradiation in mouse models with this genetic background.^3^

### Histopathologic Analysis

After euthanasia, mice underwent an extensive necropsy to dissect the trigeminal nerves, spinal nerve roots, and any other visible masses/tumors within the radiation field as well as outside as previously described.^10^ Tissues were fixed in formalin, then embedded in paraffin blocks. Five-micron thick sections were cut on a Leica rotary microtome and stained with haematoxylin and eosin (H&E). H&E slides were scanned and visualized using Aperio Imagescope. Scanned histopathologic images were reviewed in a blinded manner by A.H.

### Statistical Analysis

Data were plotted and statistical tests were performed using Prizm (Graphpad). Comparisons of mouse survival after irradiation between groups were performed using log-rank tests. Comparisons of the tumor proportions between groups were performed using Chi-square tests.

## Results

### Spinal Irradiation of Benign Plexiform Neurofibromas Reduces Overall Survival

Plexiform neurofibromas are benign neoplasms that can cause debilitating symptoms and the role of radiotherapy is controversial. Also complicating overall management is that individuals with NF1 are susceptible to de novo radiation-induced malignancies^7^ related to mutagenesis of normal tissues. In prior work, we found that focal fractionated irradiation in the *Nf1* mutant background was associated with a significant reduction in overall survival^8,9^ occurring in a dose-dependent manner. In the current study, we utilized the previously reported dosing-schema, now directed to the thoraco-lumbar spine of mice (Supplementary Figure S1).

*Nf1* periostin-Cre mice develop extensive spinal plexiform neurofibromas by 5 months of age.^10^ These plexiform neurofibromas rarely spontaneously transform to higher grade MPSNTs, however transformation can be promoted by loss of CDKN2A^10^ as well as p53 knockdown.^11^ These models recapitulate the spontaneous PN to MPNST progression that occurs in approximately 10% of individuals with NF1.^12^

Five-month-old mice were randomized to radiation doses of 0 Gy, 3 Gy given daily over 5 days, and 3 Gy given daily over 10 days, one fraction per day, 5 days per week, similar to clinical practice. Radiotherapy was directed to the thoraco-lumbosacral spine via symmetrically weighted opposed lateral beams delivering 3 Gy to the midplane. Animals were monitored for up to 12 months for signs of illness such as progressive weakness, loss of ambulation, and weight loss.

**Figure 1.**
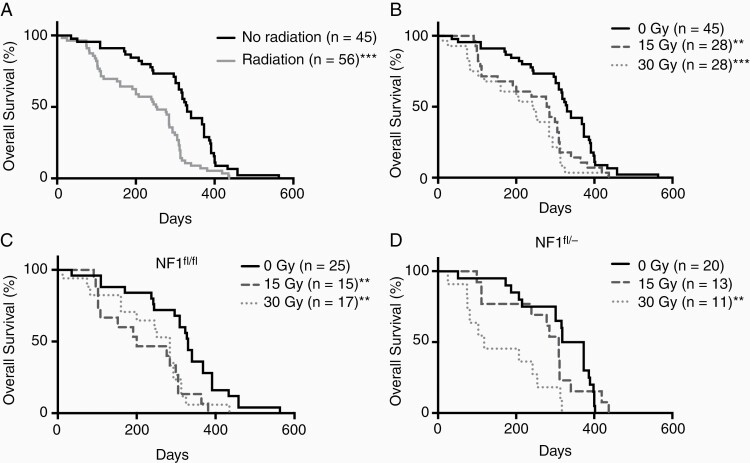
The overall survival of mice following SI. Log-rank tests were used to evaluate statistical significance between curves (*P < .05, **P < .01, ***P < .001). (A) SI decreased overall survival (OS) (all irradiated compared to unirradiated controls (*P* < .001)). (B) SI at 15 and 30 Gy were associated with a significant reduction in survival compared to no radiation controls (*P* < .01 and *P* < .001, respectively). (C) In *Nf1*^*fl/fl*^ mice, SI reduced OS at 15 Gy (*P* < .01) and 30 Gy (*P* < .01) compared to unirradiated controls. (D) OS of *Nf1*^*fl/-*^ mice was decreased following SI at 30 Gy (*P* < .01).

SI significantly decreased overall survival in the entire cohort (median survival 330 and 252.5 days for controls and radiated mice, *P* < .01 log-rank test, Figure 1A). Survival after irradiation was dose dependent, as 15 and 30 Gy was associated with significantly lower overall survival compared to 0 Gy controls (Figure 1B, median survival 330, 281, and 243.5 days for control 15 and 30 Gy groups, *P* < .01 log-rank test). The median survival of mice irradiated at 15 Gy was lower than 30 Gy but statistical significance was not achieved (*P* = .25 log-rank test).

Tumor microenvironment impacts tumorigenesis via multiple potential mechanisms.^13^ To assess whether different tumor microenvironments affect the overall survival of mice receiving different doses of SI, we analyzed the overall survival of mice on the basis of germline heterozygosity of the *Nf1* gene. Our prior models tested susceptibility to radiation-driven tumorigenesis in genetic backgrounds that were either uniformly wildtype or uniformly *Nf1*^*+/*^.^8,9^ In both models, Schwann cells are *Nf1* null, however, the cells constituting the microenvironment and immune system are either wildtype or *Nf1*^*+/-*^, enabling a test of whether post-radiation changes in the PN are influenced by the genotype of non-PN intrinsic cells such as the microenvironment and potentially systemic effects.

Survival analyses of *Nf1*^*fl/f*^ mice (Figure 1C) showed a significantly worse survival following SI at 15 and 30 Gy compared to unirradiated controls (*P* < .01, median survival 330, 200, and 284 days for control, 15 and 30 Gy). In contrast, SI at 30 Gy decreased the overall survival of male *Nf1*^*fl/-*^ mice compared to controls (*P* < .01), but not at 15 Gy (Figure 1D, median survival 345, 309, and 118 days for control, 15 and 30 Gy). Male *Nf1*^*fl/-*^ mice receiving 30 Gy experienced the worse survival (median survival 118 days). The wildtype *Nf1* tumor microenvironment demonstrated greater radiosensitivity at the lower radiation dose compared to the heterozygous *Nf1* tumor microenvironment, although the most severe reduction in survival was observed in *Nf1*^*fl/-*^ mice.

### Irradiated Spinal Plexiform Neurofibromas Develop Increased Cellularity and Higher-Grade Histologic Features

NF1-associated nerve sheath tumors may pathologically be classified based on their histopathological features along a spectrum from plexiform neurofibroma to high-grade MPNST, using recently developed consensus criteria.^10^ Observations from patients with NF1 suggest that these criteria translate to clinical outcome.^14^ Specifically, plexiform neurofibromas are characterized by a diffusely enlarged nerve with thin, wavy nuclei and a myxoid to collagenous matrix. Cellular neurofibroma (CNF) retains NF architecture, but shows hypercellularity without any other atypical features. Atypical neurofibromatous tumor of uncertain biologic potential (ANNUBP) possess 2 of 4 key features, including cytologic atypia, loss of neurofibroma architecture, hypercellularity, as well as mitotic index between 1 mitotic figures (mf)/50 and 3 mf/10 high power fields (HPFs). Low-grade MPNSTs have features of ANNUBP with mitotic index of 3–9 mf/10 HPFs without necrosis. High-grade MPNSTs have a mitotic index of 10 mf/10 HPFs or 3–9 mf/10 HPFs combined with necrosis.^15^

**Figure 2.**
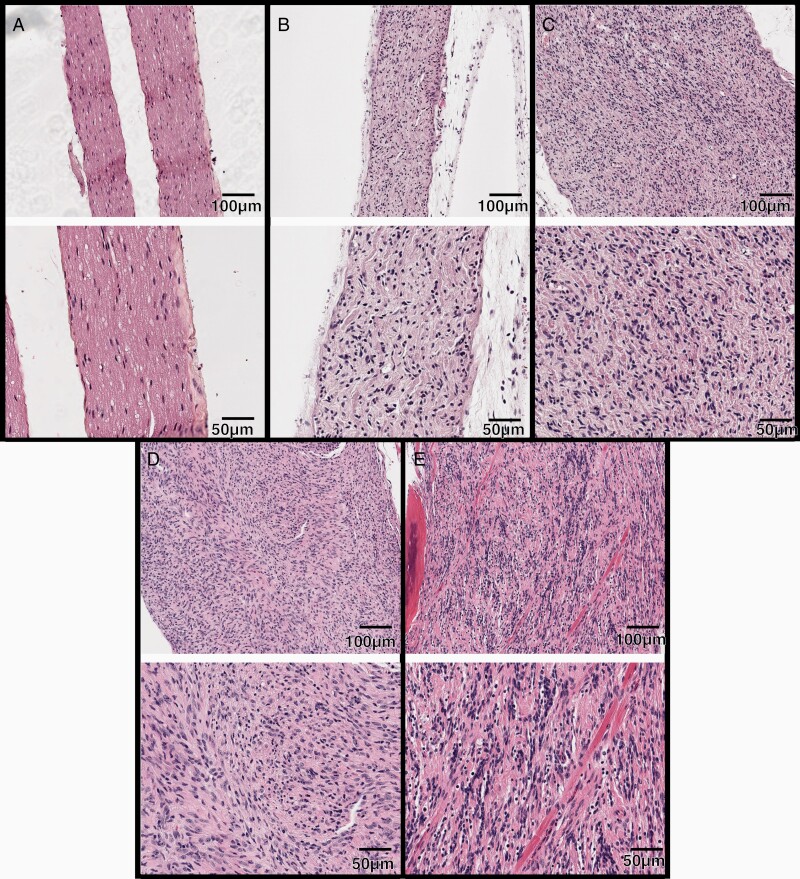
Progressively increased atypia and hypercellularity within irradiated PNs after SI. H&E-stained sections of normal nerve and nerve sheath tumors arising after focal spinal irradiation. 10X magnification (top) and 20× (bottom) magnification images shown. (A) normal nerve from a wildtype mouse. (B) PN showing diffuse enlargement and replacement of the nerve fascicle by spindle cells and collagen. (C) CNF with more prominent hypercellularity. (D) ANNUBP. (E) Low-grade MPNST infiltrating skeletal muscle.

Formalin-fixed paraffin-embedded spinal nerve samples (cervical through sacral) were analyzed from 48 mice. As is typical of these mouse models, widespread plexiform neurofibromas were visible throughout the spinal nerve tissues, with the majority of neoplasms being PNF (30/48), followed by cellular neurofibroma (CNF, 15/48), atypical neurofibromatous neoplasm of uncertain biologic potential (ANNUBP, 1/48) and low-grade MPNST (2/48). All of these histologies localized to spinal nerves (Figure 2).

**Figure 3.**
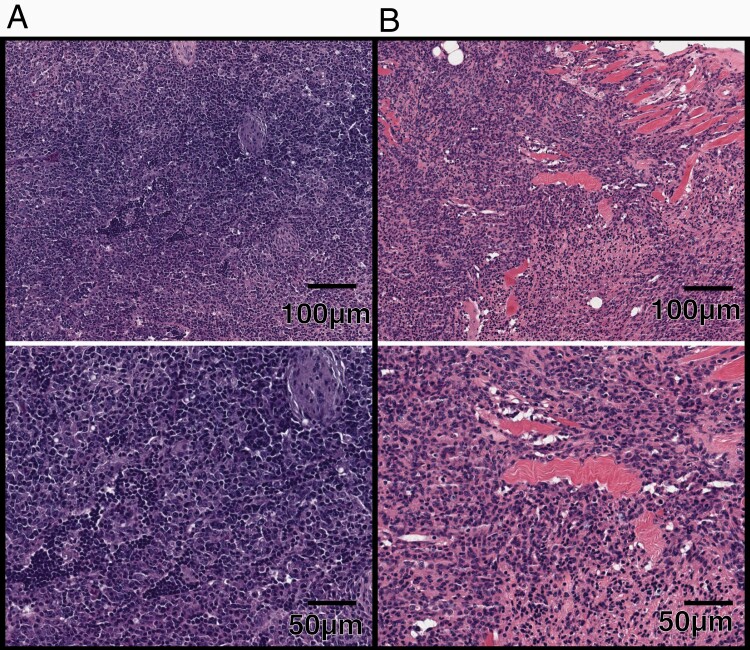
H&E-stained sections of masses developed outside of spinal irradiation field. (A) 10× (top) and 20× (bottom) magnification of a high-grade lymphoma. (B) 10× (top) and 20× (bottom) magnification of an ulcerated cutaneous neurofibroma. (C) 10× (top) and 20× (bottom) magnification of a follicular infundibular cyst. (D) 10× (top) and 20× (bottom) magnification of an undifferentiated malignant neoplasm entrapping the epididymis.

A small minority of animals developed neoplasms outside the irradiated spinal field; 3 neoplasms arose outside of the spinal irradiation field, including 2 mice with high grade lymphoma and an undifferentiated malignant neoplasm entrapping the epididymis (Figure 3). This limited number of malignancies outside of the spinal irradiation field is consistent with irradiation delivered focally to the spine (Supplementary Figure S1). Altogether, the presence plexiform neurofibroma and its more aggressive histologies in the majority of mice, as well as the relatively the low incidence of neoplasms outside of the spinal irradiation field served as an early indication that that the deaths and signs of illness in the majority of mice were associated with the presence of plexiform neurofibroma and its more aggressive histologies.

**Figure 4.**
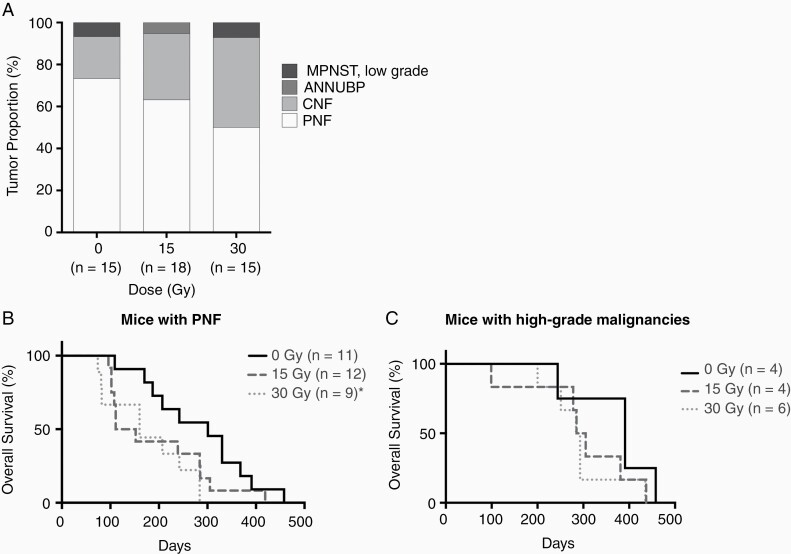
Tumor proportions and the overall survival of mice following SI grouped based on tumor diagnosis. (A) Bar plot of tumor proportion (Y axis) for each doses of SI (X axis). (B) X axis showed days following SI, Y axis showed overall survival (%). Log-rank tests were used to show statistical significance between curves. Statistical significance (*P* < .05) was shown by asterisk (*). The overall survival of mice with PNF after SI at 30 Gy was significantly lower than controls (*P* = .04), but not at 15 Gy (*P* = .08). (C) The overall survival of mice diagnosed with high-grade malignancies was not statistically different between groups.

To understand the relationship between increasing doses of IR and malignant transformation, we compared the PN-associated histologies developing after escalating doses of SI. The frequency of PNs decreased as SI dose increased and correspondingly the frequency of higher-grade histologic changes increased (Figure 4A, *P* = .43 Chi-square test). Although statistical significance was not achieved, comparison of survival between unirradiated mice with and without diagnosis of high-grade PN indicated that higher grade malignancies in unirradiated mice likely developed due to old age (median survival 391 vs 301 for unirradiated mice with vs without higher-grade PNs). Among mice developing PNF, mice receiving 30 Gy SI experienced reduced survival compared to controls (Figure 4B, median survival 301 and 160 for 0 and 30 Gy, *P* = .01 Log-rank test). There was no difference in the overall survival of male mice with high-grade malignancies receiving different doses of SI (Figure 4C, median survival 391, 295, and 288 for 0, 15, and 30 Gy, respectively, *P* = .32, Log-rank test) due to relatively low incidence of high-grade malignancies. Despite not reaching statistical significance, these data provided a preliminary indication that fractionated IR may promote progressive neoplastic changes in *Nf1* mutant PNs.

## Discussion

In individuals with tumor predisposition syndromes and germline mutations, concerns regarding treatment-induced progressive transformational changes arise during medical management, however, due to potential ethical issues and lack of uniform radiation dosing this question cannot be answered with clinical studies. Experimental models allow for controlled studies and testing of uniformly applied radiation dosing, which are difficult in the clinical setting.

In this study, we successfully employed a unique approach utilizing Schwann cell-specific *Nf1* knockout mice serving as a model of benign spinal plexiform neurofibromas and fractionated-focal spinal irradiation regimen, to investigate the effects of ionizing radiation to the disease process of NF1-driven tumorigenesis. This experiment specifically evaluates overall survival and provided a preliminary indication of malignant transformation of PNs to more aggressive tumor histologies. These findings provide experimental information that supports careful consideration regarding the role for radiotherapy in the management of PNs in NF1 patients.

Prior work experimentally comparing PN progression after bone marrow transplantation demonstrated that the *Nf1*^*+/-*^ tumor microenvironment specifically promotes progression of PN.^16^ The present study and survival differences between genetic backgrounds are also consistent with a tumor extrinsic mechanism, possibly originating in the tumor microenvironment, contributing to the worse survival of male *Nf1*^*fl/-*^ mice receiving 30 Gy SI. Genetic background effects are highly relevant for patients with germline-mediated diseases, who, similar to our patient, may be at heightened risk for more severe pathologic processes in response to therapies.

Interestingly, our mouse models did not develop typical radiation-induced second cancer histologies such as sarcomas but instead developed histologically aggressive features within existing PNs only, reflecting progression originating from the PNs themselves, rather than adjacent normal tissues. This aspect of our model suggests that among the risks faced by individuals with NF1, progression of benign irradiated disease may represent a greater hazard than induction of a normal-tissue-derived secondary cancer.

A limitation of this study is the relatively low numbers of higher-grade tumor histologies found following exposure to IR in this model. Higher number of biological replicates in future studies are needed to better establish an association between the overall survival, tumor aggressiveness, and radiation doses. Further, this study did not investigate the effects of radiation and genetic background at molecular levels, which in future studies may provide more information on mechanistic relationship between radiation dose, different germline background, and tumor aggressiveness.

In summary, our data provide a preliminary evidence that radiation exposure may drive the transformation of PN into tumors with increasingly worrisome histologies and that may lead to reduced survival. Our data also indicate that the germline *Nf1* status influences this response. Possible contributing mechanisms may involve the microenvironment and immune system, and point to the role of tumor-extrinsic processes mediating tumor progression and radiation response. Future studies focusing on the mechanistic relationship between radiation doses and tumor aggressiveness, as well as the influence of germline backgrounds, may provide clearer insights on the biology of radiation-induced malignant transformation in NF1 context.

## Supplementary Material

vdab063_suppl_Supplementary_Materials

## Notes

**Authorship Statement.** Concept establishment and study design: D.W.C. and J.L.N. Development of mouse irradiation methodology: M.M. Mouse irradiation and experimentation: A.E.S., H.C.S., and W.K.B. Analysis and interpretation of data: D.L., A.H., M.D., and J.L.N. Manuscript writing: J.L.N. and D.L. Final approval of manuscript: D.L., A.E.S., W.K.B., M.M., H.C.S., M.D., A.E.H., D.W.C., and J.L.N.
