# Supplementary material for: Irradiation of Nf1 mutant mouse models of spinal plexiform neurofibromas drives pathologic progression and decreases survival
Source: Neurooncol Adv. 2021 Apr 23;3(1):vdab063. doi: 10.1093/noajnl/vdab063 (PMC8193912; doi:10.1093/noajnl/vdab063)

**Supplementary Figure Captions**

**Supplementary Figure S1. A schematic of mouse focal irradiation to the spine.** Mice were place between two blocks and on a wedge to linearize spine and allow for a selective, focal irradiation to the spine.

Supplementary Figure S1


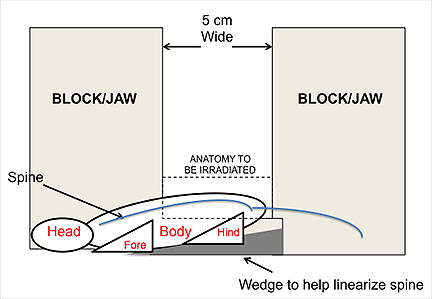

Supplement: vdab063_suppl_Supplementary_Materials [file vdab063_suppl_supplementary_materials.zip › vdab063_suppl_Supplementary_Materials.docx]
