# Supplementary figures and images for: Irradiation of Nf1 mutant mouse models of spinal plexiform neurofibromas drives pathologic progression and decreases survival
Source: Neurooncol Adv. 2021 Apr 23;3(1):vdab063. doi: 10.1093/noajnl/vdab063 (PMC8193912; doi:10.1093/noajnl/vdab063)

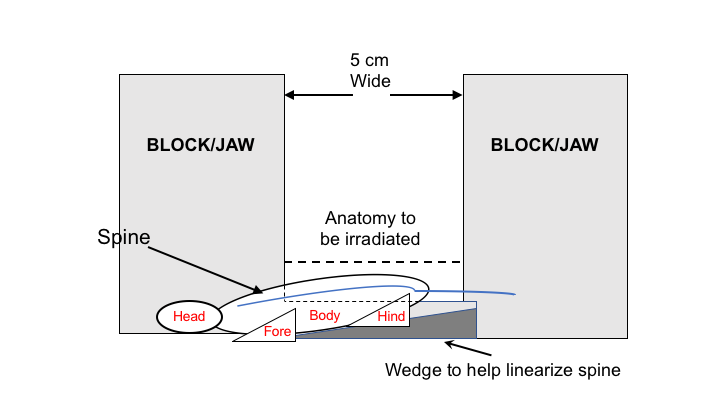

Supplement: vdab063_suppl_Supplementary_Materials [file vdab063_suppl_supplementary_materials.zip › vdab063_suppl_Supplementary_Figure_S1.tiff]
